# 5′ Rapid Amplification of cDNA Ends (5′ RACE)-Based Targeted RNA Sequencing Assay for the Analysis of Clinically Relevant Alterations in Lung Cancer Patients

**DOI:** 10.3390/ijms27167226

**Published:** 2026-08-13

**Authors:** Natalia V. Mitiushkina, Elena V. Preobrazhenskaya, Aleksandr A. Romanko, Anna D. Shestakova, Rimma S. Belova, Tatiana Y. Velyukhova, Yana I. Tretyakova, Natalia A. Firsova, Ekaterina A. Nalivalkina, Vladislav I. Tiurin, Evgeny N. Imyanitov

**Affiliations:** 1 Department of Tumor Growth Biology, N.N. Petrov Institute of Oncology, Saint Petersburg 197758, Russia; 2 Department of Medical Genetics, Saint Petersburg State Pediatric Medical University, Saint Petersburg 194100, Russia

**Keywords:** targeted RNA sequencing, 5′ RACE, NSCLC, targeted therapy, fusion, rearrangement

## Abstract

Next-generation sequencing (NGS) is currently regarded as a preferable method for detection of actionable alterations in non-small cell lung carcinomas (NSCLCs). RNA-based NGS is particularly efficient in detection of gene fusions and allows for mRNA expression analysis of relevant genes. This study describes a novel library preparation pipeline for targeted RNA sequencing, which is based on the 5′ rapid amplification of the cDNA ends (5′ RACE) and anchored multiplex PCR. The NGS panel was designed for the analysis of 15 genes relevant to NSCLC therapeutic decisions (*ALK*, *BRAF*, *CD274* (*PD-L1*), *EGFR*, *ERBB2* (*HER2*), *KRAS*, *MET*, *NRAS*, *NRG1-2*, *NTRK1-3*, *RET* and *ROS1*). The validation against PCR was performed for 168 NSCLC samples. The NGS assay successfully detected all 85 mutations previously identified by PCR. It also confirmed the absence of tested mutations in 82 out of 83 PCR-negative samples. The only discordant case was subsequently analyzed by digital droplet PCR and demonstrated low fraction of the mutated allele. The newly designed NGS panel was subsequently used for the analysis of 272 carcinomas from young (≤50 years old) patients with no driver mutations identified by PCR tests or with failed PCR analysis. NGS was successful in 240 (88.2%) cases, including 35 (64.8%) PCR-failed samples. Driver mutations were detected in 55 tumors, including three previously unreported fusions (*PAPLN::ALK*, *CD55::ROS1* and *FKBP15::RET*). We conclude that 5′ RACE-based RNA sequencing is a viable approach for NSCLC molecular testing.

## 1. Introduction

Clinical management of non-small cell lung cancer (NSCLC) requires testing for a wide range of molecular alterations. These genetic events include “common” and “rare” *EGFR* mutations, *ALK*, *ROS1*, *NTRK1-3* and *RET* fusions, *MET* exon 14 skipping mutations, selected substitutions in *KRAS* and *BRAF* genes, *HER2*-activating alterations, and *PD-L1* expression [1,2,3]. Recently, *NRG1/2* fusions were recognized as potentially targetable events in the lung cancer setting [3,4,5]. Single-gene consecutive testing is becoming increasingly labor-intensive and inefficient as the landscape of molecular-guided NSCLC treatments evolves. Although it is beyond a doubt that next-generation sequencing (NGS) is a preferable method for NSCLC profiling, the choice of suitable platform remains complicated. The majority of commercial NGS-based tests cover a few hundred actionable and potentially actionable genes, with only a tiny fraction of those being relevant to NSCLC; these panels require middle-to-high throughput instrumentation, which is not feasible for hospital-based laboratories. More importantly, currently utilized diagnostic NGS assays are usually DNA-based, i.e., they are deficient in detecting tyrosine kinase rearrangements. RNA-based sequencing approaches have significant advantages, as they outperform DNA-based platforms concerning detection of actionable gene fusions [6,7]. RNA-based sequencing allows reliable identification of activating oncogenic mutations. Interestingly, it was reported that the allele, which carries an activating mutation, often shows higher expression compared to the wild type copy of the same gene [8]. Thus, such mutations can be even more easily detected with the use of RNA vs. DNA sequencing, especially in clinical samples with low tumor cell content. It is not possible to test directly for gene amplifications by RNA-based methods. However, functional amplifications are supposed to result in increased mRNA expression of a given gene, and the amount of specific transcripts can be measured using targeted RNA-based NGS. Moreover, overexpression of particular genes, even in the absence of amplification, can sometimes be recognized as a targetable event [9]. Regarding the PD-L1 testing in NSCLC, immunohistochemistry (IHC) remains the standard approach, though the parallel analysis of protein and mRNA expression levels showed rather good concordance in a number of investigations [10,11].

In this study, the libraries for targeted RNA sequencing were constructed using a newly developed method, which utilizes 5′ rapid amplification of the cDNA ends (5′ RACE) and anchored multiplex polymerase chain reaction (PCR) techniques [12,13]. Unlike commonly used approaches, such as Archer Dx or hybridization-based targeted NGS [7,13], RACE-based targeted RNA sequencing does not require adapter ligation, and only two enzymes, namely Moloney Murine Leukemia Virus (M-MLV) reverse transcriptase and Taq DNA polymerase, are necessary for library construction. We have previously published protocols for 3′ RACE RNA sequencing; this approach is suitable for the analysis of *FGFR* gene family rearrangements, given that the unknown translocation partners are located at the 3′ end of the respective fusion transcripts [14,15]. However, clinically relevant translocations in NSCLC (e.g., fusions involving *ALK*, *ROS1*, *NTRK1-3* and *RET* genes) result in other types of chimeric transcripts, where unknown gene partners are located upstream of the involved kinase. Thus, the analysis of actionable gene rearrangements in NSCLC requires an alternative approach, and 5′ RACE-based NGS can become a method of choice in such situations.

The frequency of actionable translocations is known to be particularly high in early-onset NSCLCs [16,17]. In this study, we analyzed NSCLC tumor samples from young patients (≤50 years old) with negative PCR testing results or failed PCR analysis to identify missed and rare actionable events specific to such patients, as well as to evaluate the clinical utility of the newly developed 5′ RACE-based NGS panel.

## 2. Results

### 2.1. Validation of the 5′ RACE-Based Targeted RNA Sequencing Panel

**Figure 1:**
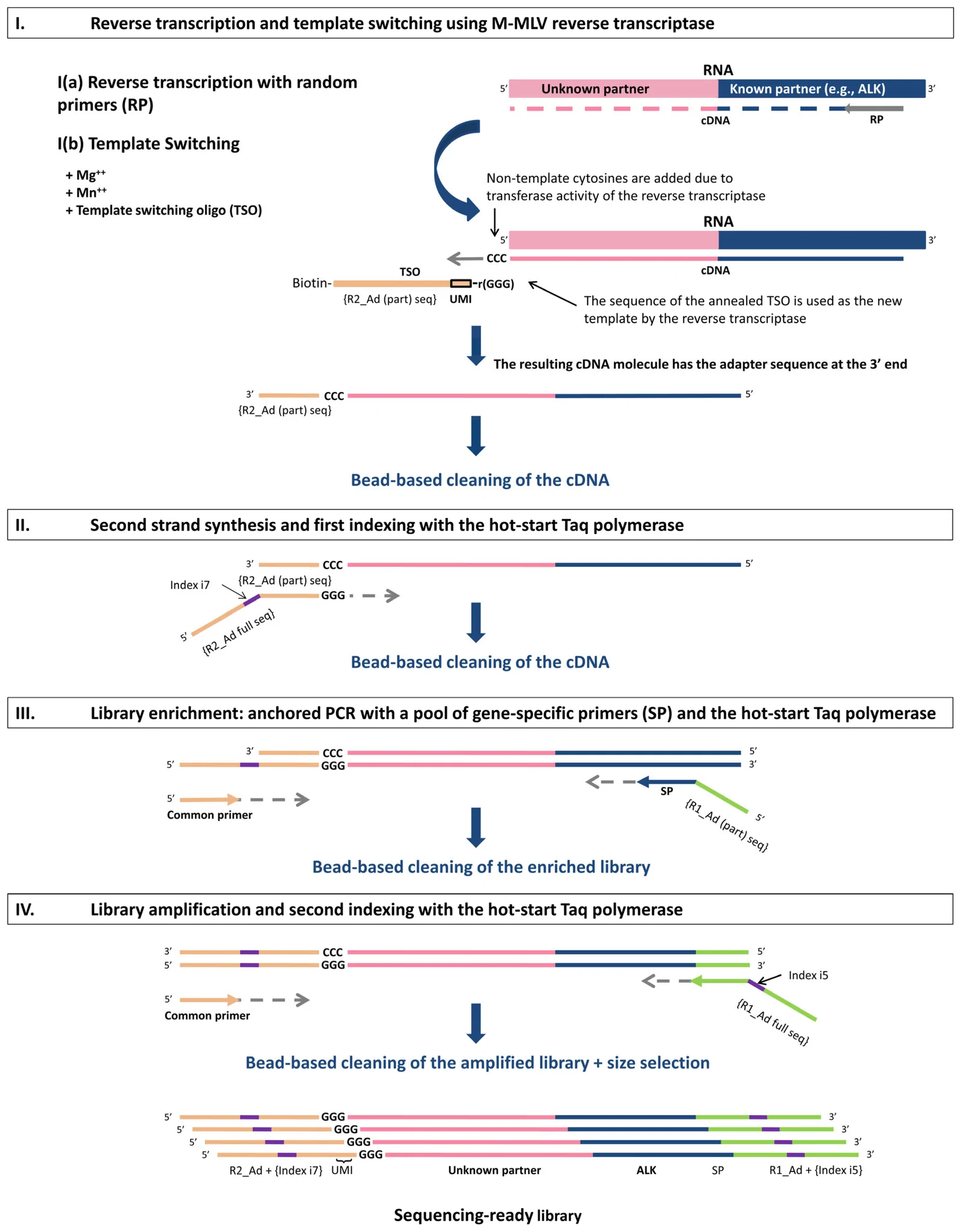
5′ RACE-based targeted RNA sequencing library preparation pipeline. Abbreviations: RP—random primers; R1_Ad, R2_Ad—NGS adapter sequences required for the hybridization with flow cell, bridge amplification and generation of paired reads 1 (R1) and 2 (R2), respectively; seq—nucleotide sequence; SP—gene-specific primer; TSO—template switching oligonucleotide; UMI—unique molecular identifier.

The detailed protocol for 5′ RACE-based targeted RNA sequencing is described in Section 4. Figure 1 shows the principle of the method. Briefly, the library preparation pipeline consisted of four main steps: (I) reverse transcription (RT) reaction with the M-MLV reverse transcriptase, which was immediately followed by the template switching reaction; (II) synthesis of the second cDNA strand and indexing using Taq DNA polymerase; (III) library enrichment with multiplex anchored PCR; (IV) amplification, second indexing and size selection of the NGS library fragments.

As a first step, we analyzed the performance of the newly designed NGS panel. The results obtained by the 5′ RACE-based targeted RNA sequencing were compared with data produced by PCR assays in a series of consecutive lung cancer patients. The markers analyzed by both methods included activating mutations in the *EGFR*, *KRAS*, *NRAS* and *BRAF* (exon 15) genes, rearrangements involving *ALK*, *ROS1*, *RET* and *NTRK1-3* tyrosine kinases, *ERBB2* (*HER2*) exon 20 insertions and *MET* exon 14 skipping mutations. Of the 186 tested samples, two (1.1%) failed the NGS-based analysis, while another 16 (8.6%) were determined to have low coverage. Among the 168 samples with good-quality NGS results, excellent concordance with PCR data was observed: 85 tumors were found to be mutation-positive, and 82 tumors were classified as being wild type for all tested alterations by both PCR and NGS (Table S1). In a single remaining case, a *KRAS* p.G12D mutation was found by RNA NGS with relatively low mutant allele prevalence (~6%), in a sample that was previously determined to be wild type by PCR. The presence of this mutation was further confirmed using digital droplet PCR, with 2% of the mutant alleles found at the DNA level. Variants successfully identified by the novel 5′ RACE-based targeted RNA assay included fusions involving *ALK* (*n* = 6), *RET* (*n* = 2) or *ROS1* (*n* = 2) genes, *BRAF* V600E mutations (*n* = 2), *EGFR* point mutations in exons 18–21 (*n* = 13), *EGFR* exon 19 deletions (*n* = 7) and exon 20 insertions (*n* = 3), *HER2* exon 20 insertions (*n* = 4), *KRAS* mutations in codons 12 (*n* = 36), 13 (*n* = 3) and 61 (*n* = 1), *MET* exon 14 skipping (*n* = 6), and *NRAS* codon 61 mutation (*n* = 1).

In seven of eight samples with low NGS coverage, which were determined to be mutation-positive by PCR analysis, the respective alterations were still successfully identified, including instances of *KRAS* codon 12 mutations (*n* = 2), L858R substitution (*n* = 1), exon 19 deletions (*n* = 2) and insertion in exon 20 (*n* = 1) of the *EGFR* gene, and the insertion in exon 20 of the *HER2* gene (*n* = 1). However, the NGS assay failed to identify *EML4::ALK* (E6;A20) fusion in one such sample with low coverage.

### 2.2. Analysis of Lung Carcinomas from Young Patients

**Figure 2:**
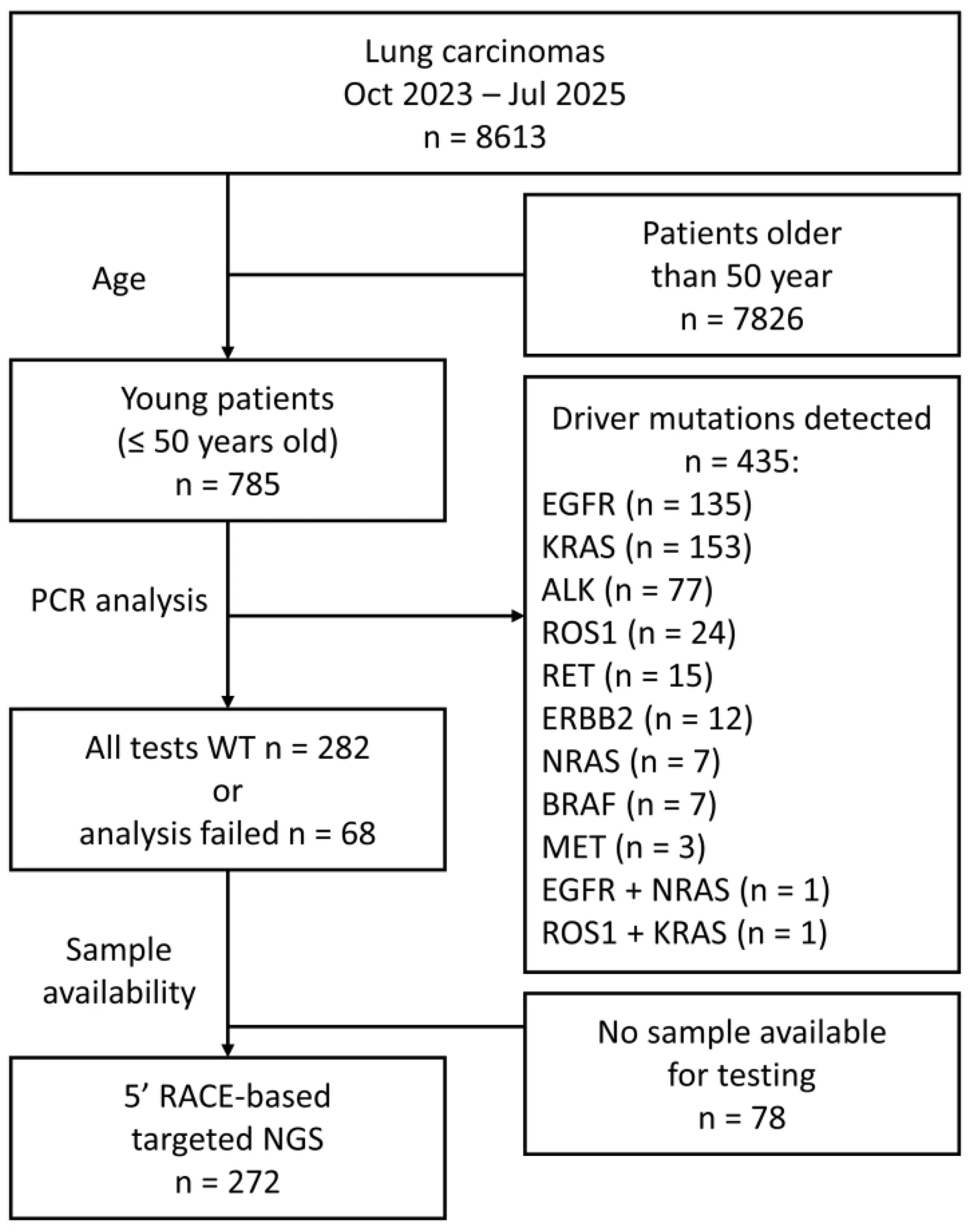
Flowchart describing patient selection for the early-onset NSCLC group.

To explore the ability of the newly designed 5′ RACE-based NGS panel to identify rare or previously missed genetic alterations, 272 tumor samples from early-onset (≤50 years old) NSCLC patients were subjected to the analysis. Only patients with no driver alterations identified by PCR or with unsuccessful PCR analysis were included (Figure 2). The characteristics of patients and tumors are shown in Table 1. NGS analysis was successfully done for 240/272 (88.2%) patients, while in 19 cases data were considered unreliable due to low coverage, and samples from other 13 patients failed the analysis. Among 54 tumor samples, which failed some or all PCR tests, 35 (64.8%) could be successfully analyzed by NGS, while for another 10 samples low coverage data were obtained, and the remaining nine specimens failed the NGS analysis. It is of note that, for the analysis of tumors from young NSCLC patients, the primer panel was extended to allow for identification of rare events, which were not analyzed by conventional PCR assays; in particular, it accounted for *NRG1/2* fusions, *ALK* kinase domain mutations (mostly associated with resistance to targeted therapy), *EGFR* kinase domain duplications, *BRAF* alterations with unclear actionability (point mutations in exon 11 and gene fusions), *ERBB2* S310F/Y substitutions, as well as exceptionally rare fusions involving exons 18 and 19 of *ALK* gene and exon 36 of the *ROS1* gene. Also, the panel was supplemented with three primers required for the analysis of *PD-L1* mRNA expression.

Driver mutations were discovered in 51/240 (21.3%) successfully tested samples and in 4/19 (21.0%) samples with low NGS coverage (Table 2). These included targetable *ALK*, *ROS1*, *RET* and *NTRK2* fusions in 16, six, four and one case(s), respectively. Additionally, fusions involving *NRG1* (*n* = 3) and *BRAF* (*n* = 1) genes were found (Table S2 lists all identified fusions and their genomic coordinates). Other detected variants included various activating mutations in *EGFR* (*n* = 9), *ERBB2* (*n* = 7), *KRAS* (*n* = 6) and *BRAF* (*n* = 2) genes.

Unusual chimeric gene variants or other mutations not covered by PCR tests were found in 25 cases. Strikingly, 18 samples demonstrated genetic alterations, which were included in the PCR panel but missed. In four cases, low tumor cell fraction (<15%) was the plausible explanation for false-negative PCR results. In the remaining 14 cases, the mistake of PCR may be attributed to the borderline RNA or DNA quality, tumor cell heterogeneity, low expression of a chimeric transcript, or operational errors.

Notably, 11/16 samples with *ALK* fusions detected by NGS demonstrated 5′-/3′-end mRNA unbalanced expression by PCR (with a probability of translocation ≥0.9 by at least one logistic regression model [18]); in two cases 5′-/3′-end *ALK* mRNA expression was balanced, and in the remaining three cases all PCR analyses were unsuccessful. In two *ALK*-positive cases with no signs of unbalanced mRNA 5′-/3′-end expression, the overall level of *ALK* expression was low, and the number of the chimeric junction reads was less than 20. Seven additional cases showed a high probability of translocation (>0.5) by this screening test. In six of these seven cases no *ALK* fusion variants were detected by NGS, while the single remaining case failed the analysis.

In contrast, *ROS1*-positive samples found by NGS in this study did not demonstrate unbalanced 5′-/3′-end mRNA expression of the *ROS1* gene. This is in line with results of the previous study, which demonstrated good performance of the screening test for unbalanced 5′-/3′-end mRNA expression in the case of *ALK*, but not *ROS1* gene fusions [18]. Regarding translocations involving the *RET* gene, two of four positive cases showed unbalanced 5′-/3′-end mRNA *RET* expression (using a predefined threshold of ΔCt ≥ three PCR cycles [19]).

To evaluate the performance of PCR and NGS testing, the respective failure rates were estimated. The overall PCR failure rate is detailed in the flowchart in Figure 2. Among 785 patients aged ≤50 years, 68 (8.7%) had inconclusive or failed results for at least one PCR assay, with no driver mutations identified. The NGS failure rate (i.e., failed analysis or low coverage without detectable driver mutations) differed between the PCR wild type (PCR-WT) samples (12/197, 6.1%) and those that failed prior PCR testing (16/54, 29.6%). Based on these proportions, the overall NGS failure rate across the entire cohort was estimated to be 8.1%, aligning closely with the PCR failure rate.

Overall, 46/272 (16.9%) of the included samples had low tumor cell content (≤15%). Notably, different driver mutations were identified in 11/46 (23.9%) samples with low tumor cell content and in 44/226 (19.5%) samples containing >15% tumor cells, despite higher proportion of cases, which failed the analysis or had low NGS coverage in the former group (8/46 (17.4%) vs. 24/226 (10.6%), respectively). This may be explained by higher sensitivity of RNA-based NGS versus PCR in samples with low tumor cellularity.

Higher input concentrations of nucleic acids are generally supposed to increase the NGS library diversity, reduce the number of PCR duplicates and improve NGS coverage. When developing the new library preparation pipeline, the maximum RNA input amount, which can be successfully converted to the NGS library fragments, was determined as 250 ng (see Materials and Methods). This amount of RNA was available for 191 NSCLCs, while 81 samples had a lower RNA yield. Expectedly, the probability of NGS failure or low coverage was higher in samples with low RNA input amount compared to samples, where 250 ng RNA could be taken into the reaction (15/81 (18.5%) vs. 17/191 (8.9%), respectively).

### 2.3. Comparison of the PD-L1 Expression Measurements Made with 5′ RACE-Based Targeted RNA Sequencing and Conventional Immunohistochemistry (IHC)

Both IHC PD-L1 status and NGS results of sufficient quality were available for 49 patients from the group of young NSCLC cases. Positive PD-L1 staining in >1% tumor cells was observed in 10/49 (20.4%) of these samples (Table S3). The optimal threshold for *PD-L1* mRNA normalized expression value, determined with a ROC curve analysis with IHC serving as the reference method, was 0.69 (Figure S1). With this threshold, 5′ RACE-based NGS method showed a 70% sensitivity and a 95% specificity in identifying correctly IHC PD-L1-positive samples (Table S3). The ROC analysis yielded an AUC of 0.867 (95% CI: 0.737–0.996). Given the small sample size, these findings are preliminary and require validation in larger cohorts. The false-negative results could be attributed to a low fraction (≤10%) of PD-L1-positive tumor cells in two cases and low overall tumor cell content in one case. The reasons for false-positive NGS findings observed in the two cases are not clear: these could be the consequence of tumor heterogeneity, IHC failure caused by particular posttranslational modifications of the PD-L1 protein [20], or another reason.

## 3. Discussion

In the current study, a new NGS library preparation pipeline was developed on the basis of the 5′ rapid amplification of the cDNA ends (5′ RACE) and anchored multiplex polymerase chain reaction (PCR) techniques. There are several approaches for targeted RNA sequencing that allow the identification of a wide spectrum of clinically relevant aberrations, including fusion transcripts with unknown 5′ end sequences. The 5′ RACE-based method can be regarded as one of the alternatives for an in-house design of small NGS panels for various research purposes or as the basis for new diagnostic solutions. Compared to hybridization-based methods, the proposed approach has the advantage of the relatively short library preparation time: the sequencing-ready libraries can be obtained within one or two working days, which is similar to the ArcherDX assay [13]. At the same time, the 5′ RACE-based library preparation pipeline does not require the adapter ligation; thus, there is no requirement for commercial kits, multiple enzymes or other components, which are necessary for the second strand synthesis, end repair, A-tailing and ligation itself. As only two enzymes, commonly used in genetic laboratories, are needed for the library preparation, namely M-MLV reverse transcriptase, capable of template switching, and Taq DNA polymerase, the implementation of the method is expected to be less costly compared to the alternatives. The developed method allows for analysis of activating point mutations in oncogenes, gene fusions with known 3′-end partner sequences, particular splicing events and, potentially, mRNA expression level.

**Figure 3:**
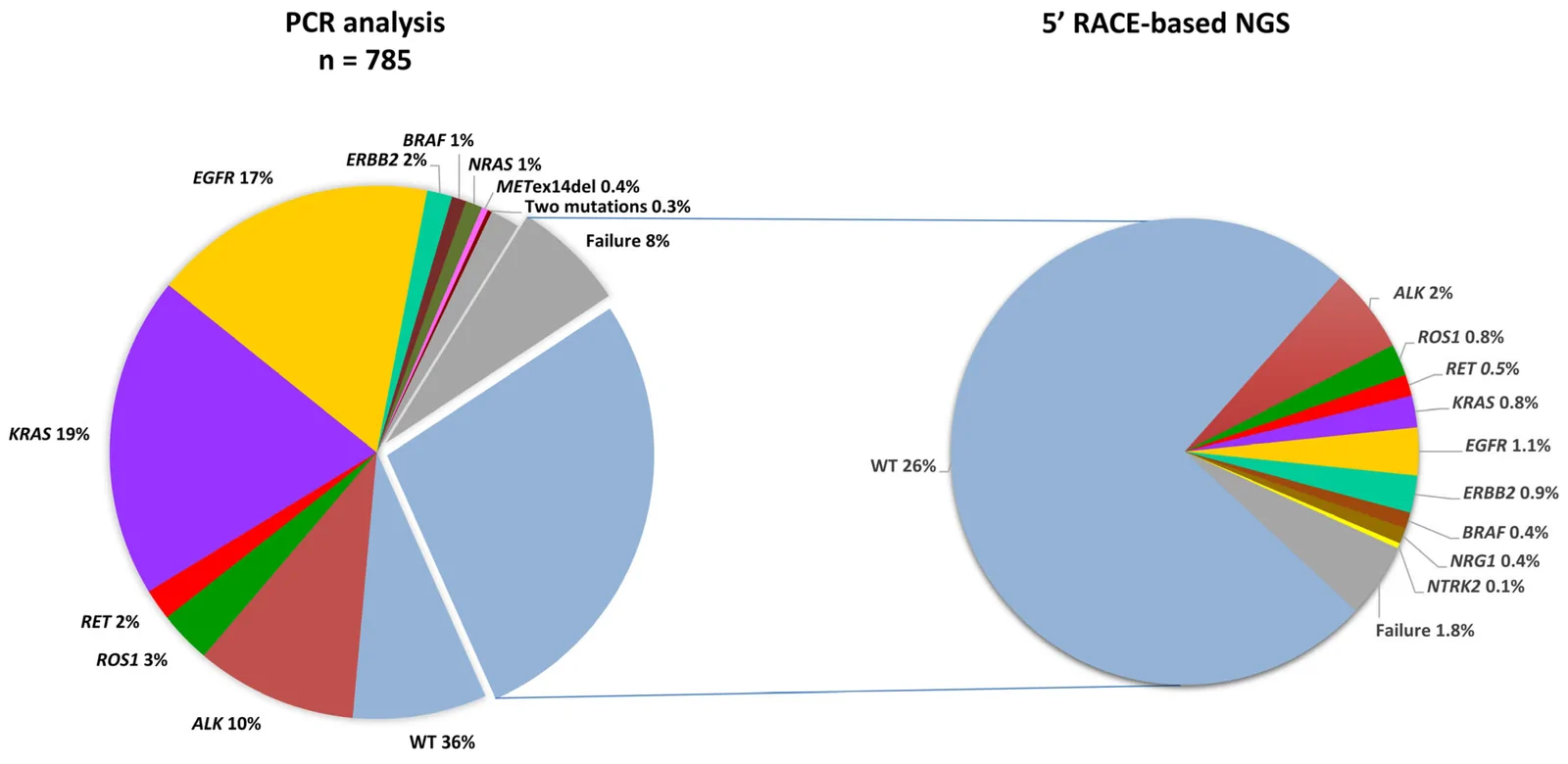
The summary of all genetic alterations found in the tumors of the early-onset NSCLC patients.

Satisfactory performance of the designed NGS panel was demonstrated through the analysis of a consecutive series of 186 NSCLC cases with available PCR testing results. Then, the extended version of the same NGS panel was applied for the study of carcinomas from young (≤50 years old) lung cancer patients. The graphical summary of all genotyping results obtained for this group of patients is provided in Figure 3. Among the 54 cases which previously failed some or all PCR tests, 35 (64.8%) were successfully genotyped with 5′ RACE-based NGS, which proves the new method is useful for the analysis of clinical tumor samples with a low amount or poor preservation of nucleic acids. Notably, targetable rearrangements appeared to be the alterations most frequently missed by conventional PCR tests. Indeed, 9.9% of all WT or failed PCR cases were found to be *ALK*, *ROS1*, *RET*, or *NTRK2* fusion-positive by NGS analysis (Figure 3). It can be calculated that 23% of cases with these clinically relevant fusions were not detected by prior variant-specific PCR tests. One reason for that is the diversity of gene fusion partners. Fusions involving unusual gene partners were found in 12/27 (44.4%) of all *ALK*/*ROS1*/*RET*/*NTRK2*-positive samples discovered only by 5′ RACE-based NGS. Three cases were present with previously undescribed fusion partners, namely *PAPLN::ALK*, *CD55::ROS1* and *FKBP15::RET*. Rare translocation variants, not yet included in our PCR panels, but previously found in NSCLC by others, included *BCL11A::ALK* [21,22], *CLIP1::ALK* [23,24], *RUFY2::RET* [13] and *SQSTM1::NTRK2* [25]. Notably, rare *CTNND1::ROS1* (C17;R35) translocation was found in two patients in this study, while previously a rearrangement involving these two genes was only mentioned once in the literature [26]. It is important to recognize recurrent rearrangements because such variants can be further incorporated in PCR and amplicon-based sequencing panels to improve the efficiency of these widely used tests. The *PABPC1::ALK* rearrangement was not previously detected in NSCLC, according to the existing literature; however, instances of this fusion were reported in an inflammatory myofibroblastic tumor and an anaplastic large cell lymphoma [27,28]. Similarly, the *ETV6::ALK* rearrangement was found previously in epithelioid fibrous histiocytoma [29] and prostate carcinoma [30], but not in NSCLC. Finally, the *TFG::ROS1* fusion, which is characteristic of inflammatory myofibroblastic tumors [31,32], was observed in the lung adenocarcinoma from one of the youngest patients in this study, a 26-year-old woman. Morphologic reassessment and additional IHC analyses (Figure S2) confirmed the diagnosis of inflammatory myofibroblastic tumor in this case, which also demonstrated the importance of molecular characterization of lung cancer tumors in young patients by NGS.

A significant proportion (11/27, 40.7%) of the targetable fusions identified through NGS in young NSCLC patients were represented by rearrangements between *EML4* and *ALK* genes, and only two of these rearrangements, *EML4::ALK* (E19;del14A20) and *EML4::ALK* (E6;A19), could not be identified by PCR due to unusual translocation breakpoints. Higher sensitivity in identifying known translocation variants by 5′ RACE-based NGS may be due to the analysis of the whole RNA sample in a single tube instead of sharing it between multiple PCRs. It may be particularly advantageous in cases with low tumor cell content and for identifying chimeric transcripts with relatively low expression levels. Also, the current study again confirmed high efficiency of the PCR assay for 5′-/3′-end mRNA unbalanced expression in screening for the translocations involving the *ALK* gene [18], with inferior performance of such tests for *ROS1* and *RET* rearrangements, as was also reported previously [18,19]. It should be noted that although the PCR test for 5′-/3′-end mRNA expression imbalance of the *ALK* gene appears to be highly reliable for the prescreening of tumors with translocation, subsequent identification of translocation by another method, be it variant-specific PCR, NGS or FISH, should be considered mandatory. Indeed, NSCLCs sometimes demonstrate *ALK* 5′-/3′-end mRNA expression imbalance in the absence of translocations, which is particularly characteristic of *KRAS*-mutated tumors [18]. Regarding the detection of *ROS1* and *RET* rearrangements, NGS appears to be more relevant as a primary diagnostic method.

Unusual fusions, involving *ALK*, *ROS1*, *RET*, *NTRK2* genes, identified in the current study are targetable with existing low-molecular weight tyrosine kinase inhibitors, which are highly effective compared to other treatment options available for NSCLC patients. Thus, identifying such fusions is of high clinical importance. Searching for rare somatic mutations through NGS analysis may help to identify patients, who can potentially benefit from new treatment options, e.g., through participation in clinical trials [33]. Other driver mutations identified in this study included one unusual substitution in the kinase domain of the *EGFR* gene (p.E711K), *EGFR* kinase domain duplication (KDD), different point mutations in the *ERBB2* gene, one *BRAF* and three *NRG1* fusions. The clinical significance of these variants is not well-established yet; however trastuzumab deruxtecan can already be prescribed to the lung cancer patients with mutations in the *ERBB2* gene [34], and zenocutuzumab was recently approved by the U.S. Food and Drug Administration (FDA) for treatment of *NRG1*-rearranged NSCLC [35]. *EGFR* KDDs are rarely observed in NSCLC; thus the available treatment-related information is scarce. Several case reports, however, documented relatively good responses of patients with these alterations to small-molecule EGFR tyrosine kinase inhibitors [36,37].

The net clinical performance of the newly developed 5′ RACE-based RNA sequencing assay deserves consideration. The standard PCR assays managed to identify therapeutic targets in 322 (41%) out of 785 early-onset NSCLC patients (*EGFR*: 136; *KRAS* G12C: 48; *ALK*: 77; *ROS1*: 25; *RET*: 15; *ERBB2*: 12; *BRAF* V600: 6; *MET*: 3). In addition, the PCR test excluded from further consideration 113 (14.4%) patients with either *KRAS* non-G12C, *BRAF* non-V600 or *NRAS* mutations. The subsequent NGS analysis identified clinically actionable findings in an additional 45 (5.7%) subjects that included gene fusions in samples with unbalanced expression, various events missed by PCR, gene alterations in PCR-failed samples or druggable genetic lesions not included in the standard PCR analysis. One may conclude that while the conventional PCR assays produced satisfactory performance in our hands, the 5′ RACE-based RNA sequencing demonstrated substantial added value and therefore can be considered for the upfront use or the analysis of PCR mutation-negative of PCR-failed NSCLCs.

While effective for fusion and mutation detection, the 5′ RACE-based NGS method showed limited concordance between *PD-L1* mRNA expression and IHC. The interchangeability between methods of PD-L1 expression analysis remains disputable [9,10,38,39]. Bulk mRNA quantification may not reflect properly the PD-L1 expression by tumor cells, and tumor cell heterogeneity may further confound the results. The data obtained in the current study demonstrated that IHC is likely to have higher sensitivity than RNA-based NGS, especially in samples with moderate level of PD-L1 expression or low tumor cell content. Currently, PD-L1 mRNA analysis by 5′ RACE-based NGS remains exploratory in nature. Unfortunately, the number of patients with available PD-L1 IHC testing results was small in this study, and larger studies are required to make conclusions regarding the ability of 5′ RACE-based NGS results to reflect properly PD-L1 status or the expression level of other therapeutic targets, such as *HER2* or *MET*, in NSCLC samples.

In conclusion, the 5′ RACE-based RNA targeted sequencing method allows for the identification of a wide spectrum of clinically important gene fusions and other activating driver mutations in lung cancer samples. This method represents a viable and affordable alternative to existing commercial panels.

## 4. Materials and Methods

### 4.1. Patients and Samples

The study involved consecutive patients, who were referred to the N.N. Petrov Institute of Oncology (St. Petersburg, Russia) between the years 2023 and 2025 for detection of *EGFR*, *KRAS*, *NRAS*, *BRAF*, *ALK*, *ROS1*, *RET*, *NTRK1-3*, *ERBB2*, and *MET* gene mutations. Although some driver mutations included in the PCR assay are not amenable to targeted therapy (e.g., mutations in the *KRAS* and *NRAS* genes, except for *KRAS* p.G12C), they are known to be mutually exclusive with other driver alterations. Therefore, samples with such mutations can generally be excluded from testing with more laborious and expensive methods (e.g., FISH or NGS), required to identify rare targetable events. The conventional diagnostics relied on the previously described PCR-based tests [18,19,39,40,41,42,43,44] (see details in Table S4). All formalin-fixed paraffin-embedded (FFPE) samples underwent pathological review and manual macrodissection to enrich for tumor cell content. Nucleic acids from FFPE tissue slices were extracted using Trizol reagent (Invitrogen, Waltham, MA, United States). The analysis of PD-L1 expression by immunohistochemistry (IHC) was done upon physician request as part of routine clinical diagnostics in a subset of patients using the Ventana PD-L1 (SP263) Assay (Ventana Medical Systems, Oro Valley, AZ, USA). The validation of a newly designed 5′ RACE RNA sequencing pipeline was done using 186 tumors from consecutive patients, which were collected within two weeks in January 2023 and successfully tested by conventional methods. Next, we considered all NSCLC patients ≤ 50 years old who had a sufficient amount of nucleic acids for RNA NGS and no driver mutation identified by PCR analysis (*n* = 272, Figure 2). This group included 54 patients with failure of PCR tests, as well as 21 patients with failure to identify rearrangement despite the presence of 5′-/3′-end mRNA expression imbalance (*ALK*: 18; *RET*: 1; *RET + ROS1*: 1; *NTRK2*: 1). The study was performed in line with the principles of the Declaration of Helsinki. Approval was granted by the Ethics Committee of N.N. Petrov Institute of Oncology (27 September 2022, #12/283).

### 4.2. Preparation of NGS Libraries Using 5′ RACE-Based Method

The concentration of RNA was first assessed by a NanoPhotometer (Implen GmbH, München, Germany). Samples with low (<50 ng/mkl) or immeasurable nucleic acid content were concentrated with vacuum centrifuge at 60 °C, and the entire sample was used for a single library preparation. RNA samples with higher concentrations were also measured with a Qubit 4.0 fluorometer (Thermo Fisher Scientific, Waltham, MA, USA), and 250 ng of RNA (or less) was taken in a reverse transcription (RT) reaction. We found that an RNA amount higher than 250 ng often resulted in failure of the template switching reaction.

The library preparation pipeline (Figure 1) consisted of the following steps:

(Ia) Reverse transcription (RT) reaction with the RevertAid H Minus Reverse Transcriptase (Thermo Fisher Scientific, Waltham, MA, USA). First, RNA was mixed with 2 μL of 5X Reaction Buffer, 2 μL of decamer random primers (50 μM), 1 μL of dNTP mix (10 mM each) and clean water to the total volume of 10 μL. To denature RNA, the mixture was heated for 3 min at 70 °C and then cooled to the room temperature. After that, the reaction was supplemented with 0.5 μL (100 U) of the reverse transcriptase and 0.25 μL (5 U) of the Thermo Scientific RiboLock RNase inhibitor. The reaction was incubated at 25 °C for 10 min and at 42 °C for 30 min before proceeding to the template switching reaction.

(Ib) Template switching reaction. The following components were mixed together and added to the RT reaction from the previous step: 2.3 μL of the template switching oligo (Biotin-GTTCAGACGTGTGCTCTTCCGATCTNNNNNNrGrGrG, where rG is riboguanosine triphosphate; 100 μM), 2 μL of 5X Reaction Buffer, 2 μL of dNTP mix (10 mM each), 1.6 μL of MgCl2 (25 mM), 1.6 μL of MnCl2 (50 mM) and 0.5 μL (100 U) of the RevertAid H Minus Reverse Transcriptase. The reaction was incubated at 42 °C for another 30 min and then at 70 °C for 10 min. After this step, the cDNA was cleaned using 1.5× volume of AmPure magnetic beads (Beckman Coulter, Brea, CA, USA) and eluted in 27 μL of clean water.

(II) Synthesis of the second cDNA strand. The composition of the reaction was as follows: 25 μL of the cleaned cDNA from the previous step, 5 μL of 10× GeneAmp PCR Buffer I (Applied Biosystems, Waltham, MA, USA), 0.5 μL (2.5 U) of TaqM polymerase (AlkorBio, St. Petersburg, Russia), 2 μL of MgCl2 (25 mM), 1.25 μL of dNTP mix (10 mM each), 2 μL of index i7 primer (CAAGCAGAAGACGGCATACGAGATXXXXXXXXGTGACTGGAGTTCAGACGTGTGCTCTTCC, where XXXXXXXX is the index sequence; 5 μM), and 14.25 μL of clean water. The reaction conditions consisted of the enzyme activation step (95 °C, 10 min), followed by 5 cycles of denaturation and synthesis steps (95 °C for 20 s, 65 °C for 1 min, 72 °C for 1 min), and final extension step (72 °C, 5 min). The reaction was cooled to 4 °C and cleaned with 1.5× volume of AmPure magnetic beads. The cDNA was eluted in 27 μL of clean water.

(III) Library enrichment with multiplex anchored PCR. The library enrichment primer panel was designed using previously developed scripts and pipelines [https://github.com/MitiushkinaNV/RACE_NGS (accessed on 26 May 2026)] [14]. The full list of primers used for enrichment is provided in Table S5. The PCR enrichment reaction contained 25 μL of the cleaned double-stranded cDNA from the previous step, 5 μL of 10× GeneAmp PCR Buffer I, 0.5 μL (2.5 U) of TaqM polymerase, 2 μL of MgCl2 (25 mM), 1.25 μL of dNTP mix (10 mM each), 2 μL of enrichment panel primer mix (0.5 μM each), 2 μL of common primer AMP-PCR2s (CAAGCAGAAGACGGCATACG; 5 μM), and 12.25 μL of clean water. The amplification program included the enzyme activation step (95 °C, 10 min) and eight PCR cycles (95 °C for 20 s, 65 °C for 10 min), followed by a final extension (72 °C, 5 min). The enriched libraries were cleaned using 1.5× volume of AmPure magnetic beads and eluted in 27 μL of clean water.

(IV) Amplification and size selection of the NGS library fragments. The PCR amplification reaction contained 25 μL of the cleaned enriched library, 5 μL of 10× GeneAmp PCR Buffer I, 0.5 μL (2.5 U) of TaqM polymerase, 2 μL of MgCl2 (25 mM), 1.25 μL of dNTP mix (10 mM each), 2 μL of the primer AMP-PCR2s (5 μM), 2 μL of the index i5 primer (AATGATACGGCGACCACCGAGATCTACACXXXXXXXXACACTCTTTCCCTACACGACGCTCTTCC, where XXXXXXXX is the index sequence; 5 μM), and 12.25 μL of clean water. The amplification program included the enzyme activation step (95 °C, 10 min), 28 PCR cycles (95 °C for 15 s, 60 °C for 30 s, 72 °C for 30 s) and a final elongation step (72 °C, 5 min).

NGS libraries were measured, pulled together and sequenced on the NextSeq 2000 (Illumina, San Diego, CA, USA) or the FASTASeq 300 (GeneMind Biosciences, Shenzhen, China) instrument.

### 4.3. Data Analysis

The bioinformatic analysis of NGS data was performed as described previously [14,15]. The open bioinformatics instruments UMI-tools (version 1.1.4) [45], STAR (version 2.7.10b) [46] and STAR fusion (version 1.15.1) [47] were used for the removal of PCR duplicates, alignment of reads and detection of gene fusions, respectively. Additionally, the Arriba (version 2.5.1) [48] was applied to targeted RNA sequencing data, which is capable not only of detecting fusions between two genes, but also of identifying intragenic rearrangements, such as kinase domain duplications (KDDs). The custom python scripts [https://github.com/MitiushkinaNV/RACE_NGS (accessed on 26 May 2026)] were used for variant calling, MET exon 14 skipping analysis and gene coverage assessment. The annotation of the called variants was done using ANNOVAR [https://annovar.openbioinformatics.org/ (accessed on 8 June 2020)] [49]. The variant calling and filtering pipeline and the respective thresholds are provided in Figure S3. The exon/codon numbers in the manuscript correspond to the Ensembl canonical transcripts [https://www.ensembl.org/info/genome/genebuild/canonical.html (accessed on 22 January 2026)], except for the ROS1 gene, whose variants were annotated using transcript ENST00000368508.7. All statistical analyses were performed in an R programming environment (R version 4.1.1) [50]. The failure of NGS-based testing was stated if the normalization factor, calculated from coverage data of the five reference genes (*CIAO1*, *HEXA*, *NEU1*, *PSEN1* and *TOR1A*), was lower than 10 [15]. The sample was considered to have low coverage if the normalization factor was lower than 50. The analysis of *PD-L1* mRNA expression was performed as described previously [15]. The reads across three exon–exon junctions (exons 2–3, 3–4 and 4–5) were summed up and normalized as a single value. Samples with low coverage were excluded from the *PD-L1* expression analysis. The ROC curve analysis was done using the R package pROC [51].

## Figures and Tables

**Table 1:** Characteristics of the young NSCLC patients and tumor samples (*n* = 272).

Characteristic		Value
Age	Years, median (range)	45 (17–50)
Sex	Male, *n* (%)	184 (67.6%)
	Female, *n* (%)	88 (32.4%)
NSCLC histology	Adenocarcinoma, *n* (%)	159 (58.5%)
	Squamous, *n* (%)	45 (16.5%)
	Large cell, *n* (%)	4 (1.5%)
	Adenosquamous, *n* (%)	1 (0.4%)
	ND, *n* (%)	63 (23.2%)
Tumor cell content	Sufficient (>15%), *n* (%)	226 (83.1%)
	Low (≤15%), *n* (%)	46 (16.9%)
PCR result	Absence of alterations (WT), *n* (%)	197 (72.4%)
	5′-/3′-end mRNA unbalanced expression (*ALK/RET/ROS1/NTRK* genes), but no fusion identified	21 (7.7%)
	Failure of DNA-based tests, *n* (%)	20 (7.4%)
	Failure of RNA-based tests, *n* (%)	10 (3.7%)
	Failure of all PCR tests, *n* (%)	24 (8.8%)

Abbreviations: NSCLC—non-small cell lung cancer; WT—wild type; ND—no data.

**Table 2:** Genetic alterations identified by 5′ RACE-based targeted RNA sequencing in young NSCLC patients.

Patient	Sex	Age	Histology	PCR Result	Alteration (Number of Chimeric Junction Reads or Mutant Allele %)	Druggable
#6	f	26	ADCA ^↓t.c.^	WT	*TFG::ROS1* (T4;R35) (122 reads)	Yes
#10	f	30	ADCA ^↓t.c.^	WT (*ALK* unbal.)	*EML4::ALK* (E19;del14A20) (1672 reads)	Yes
#15	f	33	ADCA	WT	*CDC23::BRAF* (C16del99;B9) (464 reads)	No
#17	f	34	ADCA	WT	*CCDC6::RET* (C1;R12) ^†^ (189 reads)	Yes
#19	f	34	ADCA	WT (*ALK* unbal.)	*BCL11A::ALK* (B3;A20) (357 reads)	Yes
#22	m	35	ADCA	FAIL (ALL)	*EML4::ALK* (E13;A20) ^†^ (54 reads)	Yes
#28	m	36	ADCA ^↓t.c.^	WT (*ALK* unbal.)	*PAPLN::ALK* (P14;A20) (391 reads)	Yes
#44	m	38	ADCA	WT	*CD55::ROS1* (C9;R34) (1096 reads)	Yes
#47	m	38	ADCA	WT (*ALK* unbal.)	*ETV6::ALK* (E5;A20) (253 reads)	Yes
#48	m	38	ADCA	WT	*EML4::ALK* (E6;A19) (13 reads), low NGS coverage	Yes
#49	m	38	ADCA	WT	*ERBB2* p.A775_G776insYVMA ^†^ (6%)	Yes
#52	m	39	ADCA	WT	*EGFR* exons 18–26 duplication (116 reads)	Yes
#54	f	39	NSCLC ^↓t.c.^	FAIL (DNA)	*KRAS* p.G12D ^†^ (7%)	No
#62	f	40	NSCLC ^↓t.c.^	WT	*EGFR* p.N771_H773dupNPH ^†^ (11%)	Yes
#69	f	41	ADCA	WT (*ALK* unbal.)	*EML4::ALK* (E13;A20) ^†^ (61 reads)	Yes
#70	f	41	ADCA	WT (*ALK* unbal.)	*CLIP1::ALK* (C14;A20) (151 reads)	Yes
#72	f	41	ADCA	WT	*EGFR* p.N771_H773insTQPNP ^†^ (5%)	Yes
#74	m	41	NSCLC	WT	*EGFR* p.N771delinsHH ^†^ (34%)	Yes
#76	f	42	ADCA	WT (*RET* unbal.)	*FKBP15::RET* (F25;R12) (18 reads)	Yes
#77	m	42	ADSQC	WT (*ALK* unbal.)	*EML4::ALK* (E6/E6ins33;A20) ^†^ (21/35 reads)	Yes
#78	f	42	ADCA	FAIL (DNA)	*SLC3A2::NRG1* (S2;N6) (148 reads), low NGS coverage	Yes
#79	m	42	ADCA	WT (*ALK* unbal.)	*PABPC1::ALK* (P9;A20) (2764 reads)	Yes
#84	f	42	ADCA ^↓t.c.^	FAIL (DNA)	*ERBB2* p.G776delinsVC ^†^ (12%)	Yes
#86	m	42	NSCLC ^↓t.c.^	WT	*ERBB2* p.A775_G776insYVMA ^†^ (10%)	Yes
#89	m	42	ADCA	WT	*VAMP2::NRG1* (V4;N4) (3087 reads)	Yes
#93	m	43	ADCA	WT	*EGFR* p.E711K (30%, VUS)	ND
#95	m	43	ADCA	WT (*ALK* unbal.)	*EML4::ALK* (E13;A20) ^†^ (379 reads)	Yes
#100	m	43	ADCA	WT	*ERBB2* p.D769Y (42%)	Yes
#131	m	45	NSCLC ^↓t.c.^	WT	*KRAS* p.Q61K ^†^ (15%)	No
#145	m	46	NSCLC ^↓t.c.^	FAIL (ALL)	*KRAS* p.Q61H ^†^ (22%), low NGS coverage	No
#148	m	46	ADCA	FAIL (ALL)	*SLC34A2::ROS1* (S13del2046;R32/R34) ^†^ (735/211 reads)	Yes
#149	m	46	SCC ^↓t.c.^	WT	*CTNND1::ROS1* (C17;R35) (1390 reads)	Yes
#153	f	46	NSCLC	WT	*ERBB2* p.S310Y (42%)	Yes
#165	f	47	NSCLC	WT	*CTNND1::ROS1* (C17;R35) (3045 reads)	Yes
#166	f	47	NSCLC	FAIL (ALL)	*EGFR* p.E746_A750del ^†^ (24%), low NGS coverage	Yes
#189	m	48	ADCA	FAIL (RNA)	*CD74::ROS1* (C6;R34) ^†^ (160 reads)	Yes
#195	m	48	ADCA	WT (*RET* unbal., *ROS1* unbal.)	*RUFY2::RET* (R9;R12) (28 reads)	Yes
#197	f	48	ADCA	WT	*CD74::NRG1* (C8;N6) (6364 reads)	Yes
#200	m	48	ADCA	WT	*BRAF* p.L597R ^†^ (21%)	No
#208	f	49	ADCA	WT	*RELCH::RET* (R10;R12) ^†^ (38 reads)	Yes
#210	f	49	NSCLC	WT	*EGFR* p.P772_H773insANP ^†^ (21%)	Yes
#212	f	49	ADCA	WT (*ALK* unbal.)	*EML4::ALK* (E6/E6ins33;A20) ^†^ (14/26 reads)	Yes
#213	f	49	ADCA	WT	*BRAF* p.G469A (35%)	No
#227	f	49	ADCA	FAIL (ALL)	*EML4::ALK* (E20;A20) ^†^ (1099 reads)	Yes
#228	m	49	ADCA	FAIL (DNA)	*ERBB2* p.E757K (20%, VUS)	ND
#230	f	49	ADCA	WT (*ALK* unbal.)	*EML4::ALK* (E18;A20) ^†^ (746 reads)	Yes
#234	f	49	NSCLC	WT	*EGFR* p.L747_E749del ^†^ (6%)	Yes
#235	f	49	ADCA	WT	*EML4::ALK* (E6ins33/E6;A20) (12/7 reads)	Yes
#239	f	49	ADCA	WT	*ERBB2* p.L755S (44%) + p.P761del (41%)	Yes
#245	m	50	ADCA	FAIL (DNA)	*KRAS* p.G12C ^†^ (48%)	Yes
#250	f	50	ADCA	FAIL (DNA)	*KRAS* p.G12V ^†^ (26%)	No
#261	m	50	ADCA	FAIL (DNA)	*EGFR* p.E746_A750del ^†^ (22%)	Yes
#266	m	50	ADCA	FAIL (RNA)	*SQSTM1::NTRK2* (S5;N13/N14) (70/34 reads)	Yes
#269	m	50	ADCA	FAIL (ALL)	*EML4::ALK* (E13;A20) ^†^ (57 reads)	Yes
#270	f	50	ADCA ^↓t.c.^	WT	*KRAS* p.Q61H ^†^ (13%)	No

Abbreviations and designations: m—male; f—female; ADCA—lung adenocarcinoma; SCC—squamous cell carcinoma of the lung; ADSQC—adenosquamous lung cancer; NSCLC—non-small cell lung cancer with no data on the specific histology; WT—wild type; FAIL (DNA)—failure of DNA-based PCR tests; FAIL (RNA)—failure of RNA-based PCR tests; FAIL (ALL)—failure of all PCR tests; *ALK*/*RET*/*ROS1* unbal.—5′-/3′-end mRNA unbalanced expression of the respective genes; ^↓t.c.^—low tumor cell content (≤15%); ^†^—pathogenic variants included in PCR panels; VUS—variant of uncertain significance; ND—no data.

## Data Availability

The data that support the findings of this study are available from the corresponding author upon reasonable request.

## Supplementary Materials

The following supporting information can be downloaded at https://www.mdpi.com/article/10.3390/ijms27167226/s1.

## Abbreviations

The following abbreviations are used in this manuscript:

3′ RACE	3′ rapid amplification of the cDNA ends
5′ RACE	5′ rapid amplification of the cDNA ends
AUC	Area under the curve
FDA	U.S. Food and Drug Administration
FFPE	Formalin-fixed paraffin-embedded
FISH	Fluorescence in situ hybridization
IHC	Immunohistochemistry
KDD	Kinase domain duplication
M-MLV	Moloney murine leukemia virus
NGS	Next-generation sequencing
NSCLC	Non-small cell lung carcinoma
PCR	Polymerase chain reaction
ROC	Receiver operating characteristic
RT	Reverse transcription
