# Supplementary material for: 5′ Rapid Amplification of cDNA Ends (5′ RACE)-Based Targeted RNA Sequencing Assay for the Analysis of Clinically Relevant Alterations in Lung Cancer Patients
Source: Int J Mol Sci. 2026 Aug 13;27(16):7226. doi: 10.3390/ijms27167226 (PMC13513894; doi:10.3390/ijms27167226)
Supplement: Supplementary file 1 [file ijms-27-07226-s001.zip › Supplementary Figures.pdf]

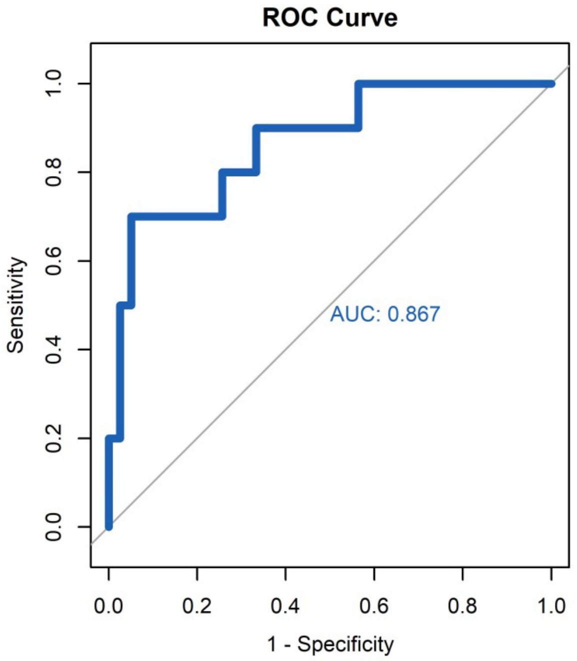

Figure S1. ROC curve obtained for the test of PD-L1 expression by 5′ RACE-based NGS, with the immunohistochemistry (IHC) PD-L1 test serving as a reference method

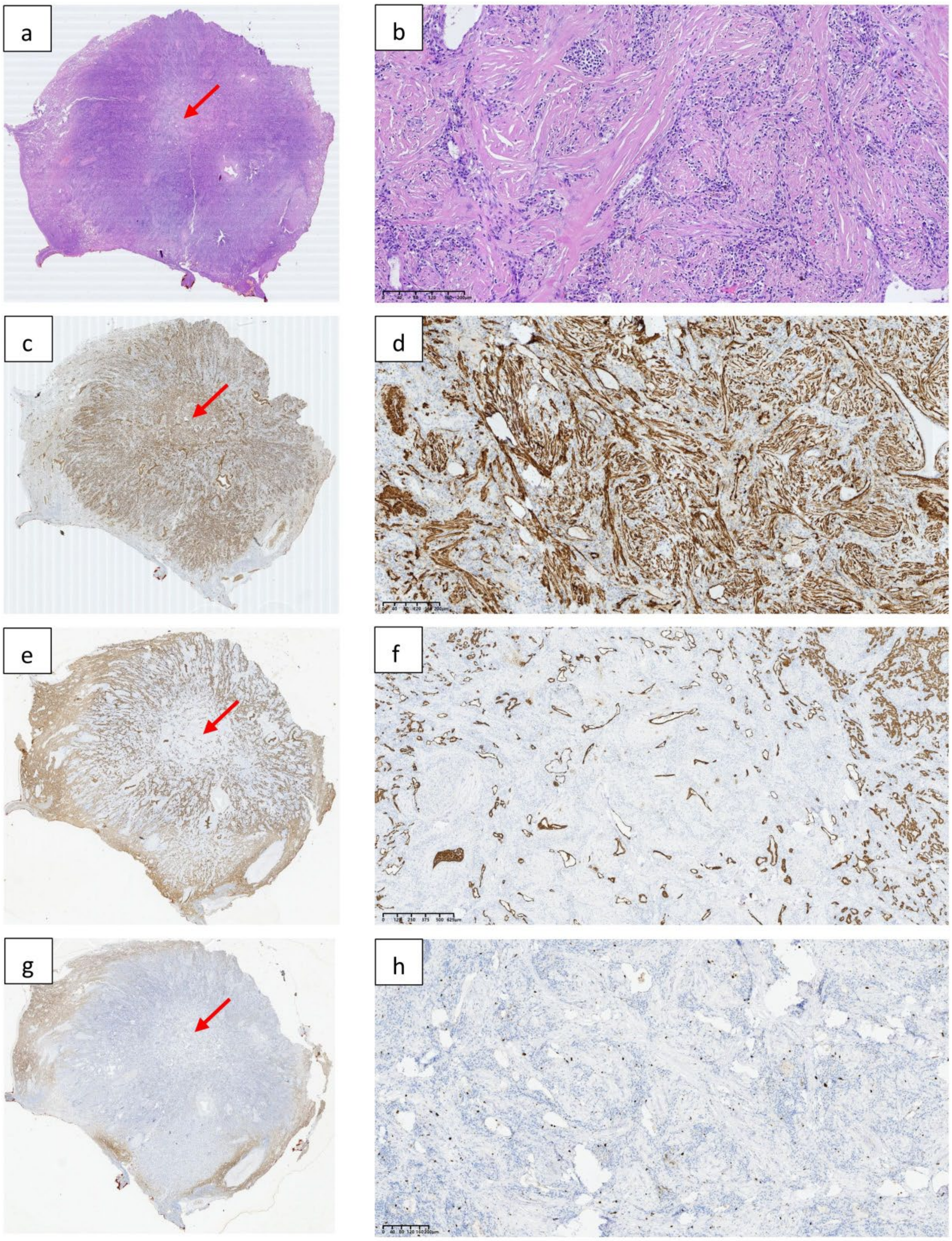

Figure S2. Confirmation of the diagnosis of inflammatory myofibroblastic tumor in a patient with *TFG*::*ROS1* fusion using immunohistochemistry (IHC): a-b, H&E (1X, 25X); c-d, IHC SMA (1A4, Dako) (1X, 25X); e-f, IHC MCK (AE1 & AE3, Cell Marque) (1X, 8X); g-h, IHC Ki67 (30-9, Ventana, Roche) (1X, 25X). The red arrows indicate the location of the tumor node.

1. Calling and filtering variants based on their allelic frequencies

Variant calling / filtering thresholds*

	SNV / indel **		Fusion / KDD	MET exon 14 skipping
	Known hotspot mutation***	Rare variant or VUS		
Mutant allele, % (minimum)	5%	10%	-	10%
Supporting/junction reads, n (minimum)	5	10	10	10

* In samples with poor RNA quality and the high error rate (e.g., due to cytosine deamination), the respective thresholds could be increased

**Only non-synonimous SNVs and non-frameshift indels were considered

***According to the literature

2. Filtering SNVs and small indels based on their population frequencies

The gnomAD v3 database [https://gnomad.broadinstitute.org] was used to annotate variant frequencies. Variants and VUSs with an overall population frequency exceeding 0.1% were excluded.

3. Filtering repeatedly occurring artifacts

Rare SNVs and indels, as well as gene fusions, were filtered out if they occurred more than twice in a single run (~96 samples).

4. Final steps

- Gene fusions were retained only if the reading frame and the tyrosine kinase domain of the gene of interest (e.g., ALK) were preserved.
- Visual inspection using the Integrative Genomics Viewer genomic browser (IGV, v2.19.2) was performed to exclude potential artifacts (e.g., read misalignment or imperfect deduplication) that could lead to erroneous calling of gene fusions or other genetic alterations.
- When necessary, known hotspot mutations were confirmed using available PCR or ddPCR assays (e.g., for variants previously missed by initial PCR screening).

Figure S3. Variant calling and filtering pipeline
